# Food additive emulsifiers and cancer risk: results from the French prospective NutriNet-Santé cohort

**DOI:** 10.1093/eurpub/ckac129.015

**Published:** 2022-10-25

**Authors:** L Sellem, B Srour, E Chazelas, C Debras, B Chassaing, I Huybrechts, F Pierre, X Coumoul, M Deschasaux-Tanguy, M Touvier

**Affiliations:** 1 Sorbonne Paris Nord University, INSERM U1153, Bobigny, France; 2 Nutrition and Cancer Research Network, Jouy-en-Josas, France; 3 INSERM U1016, Paris, France; 4 International Agency for Research on Cancer, Lyon, France; 5 Toxalim, Research Centre in Food Toxicology, Toulouse, France; 6 INSERM UMR-S 1124, Université de Paris, Paris, France

## Abstract

**Background:**

Food additive emulsifiers are found in thousands of industrial foods and may exert deleterious effects on gut microbiota and carcinogenesis according to experimental studies. However, their associations with cancer risk has not been investigated yet. This study aimed to investigate these associations in a large population-based prospective cohort.

**Methods:**

This study included 102,485 French adults from the NutriNet-Santé cohort (42.1y [14.5], 78.8% female, 2009-2021). Food additive emulsifier intakes were estimated using repeated 24h dietary records linked to brand-specific food composition databases on food additives. Associations with incident cancer risk were assessed using Multivariable Cox models.

**Results:**

3,511 incident cancer cases were diagnosed during follow-up (1,026 breast, 431 prostate, and 279 colorectal cancers). Intakes of sodium citrate (E331, HR = 1.12 [1.02-1.23], p-trend=0.009), xanthan gum (E415, HR = 1.11 [1.02-1.21], p-trend=0.02), and mono- and diglycerides of fatty acids (E471, HR = 1.17 [1.06-1.28], p-trend=0.001 and total: E471, E472a-b-c-e, HR = 1.11, [1.02-1.22], p-trend=0.02) were associated with increased overall cancer risk. Higher intakes of E331 (p-trend = 0.046), sodium stearoyl-2-lactylate (E481, p-trend=0.01), total lactylates (E481-482, p-trend=0.01), total celluloses (E460-468, p-trend=0.03), carob bean gum (E410, p-trend=0.01), and E471 (p-trend=0.006) were associated with increased overall breast cancer risk. Higher intakes of carrageenan (E407, p-trend=0.04), E415 (p-trend=0.02), and triphosphates (E451, p-trend=0.03) were associated with increased post-menopausal breast cancer risk.

**Conclusions:**

These results are the first to investigate and report direct associations between cancer risk and exposures to seven individual and three groups of food additive emulsifiers. If replicated, they may have an important public health impact, considering the omnipresence of these additives in industrial foods globally.

**Key messages:**

• This study is the first to precisely assess exposures to food additive emulsifiers in a population-based study.

• Intakes of food additive emulsifiers were associated with increased risk of cancer.

